# Intracerebroventricular Injection of Encapsulated Human Mesenchymal Cells Producing Glucagon-Like Peptide 1 Prolongs Survival in a Mouse Model of ALS

**DOI:** 10.1371/journal.pone.0036857

**Published:** 2012-06-20

**Authors:** Sarah Knippenberg, Nadine Thau, Reinhard Dengler, Thomas Brinker, Susanne Petri

**Affiliations:** 1 Department of Neurology, Hannover Medical School, Hannover, Germany; 2 Neurosurgery Foundation Providence, Providence, Rhode Island, United States of America; 3 Center for Systems Neuroscience, Hannover, Germany; University of Nebraska Medical Center, United States of America

## Abstract

**Background:**

As pharmacological therapies have largely failed so far, stem cell therapy has recently come into the focus of ALS research. Neuroprotective potential was shown for several types of stem and progenitor cells, mainly due to release of trophic factors. In the present study, we assessed the effects of intracerebroventricular injection of glucagon-like peptide 1 (GLP-1) releasing mesenchymal stromal cells (MSC) in mutant SOD1 (G93A) transgenic mice.

**Methodology/Principal Findings:**

To improve the neuroprotective effects of native MSC, they had been transfected with a plasmid vector encoding a GLP-1 fusion gene prior to the injection, as GLP-1 was shown to exhibit neuroprotective properties before. Cells were encapsulated and therefore protected against rejection. After intracerebroventricular injection of these GLP-1 MSC capsules in presymptomatic SOD1 (G93A) mice, we assessed possible protective effects by survival analysis, measurement of body weight, daily monitoring and evaluation of motor performance by rotarod and footprint analyses. Motor neuron numbers in the spinal cord as well as the amount of astrocytosis, microglial activation, heat shock response and neuronal nitric oxide synthase (nNOS) expression were analyzed by immunohistological methods. Treatment with GLP-1 producing MSC capsules significantly prolonged survival by 13 days, delayed symptom onset by 15 days and weight loss by 14 days and led to significant improvements in motor performance tests compared to vehicle treated controls. Histological data are mainly in favour of anti-inflammatory effects of GLP-1 producing MSC capsules with reduced detection of inflammatory markers and a significant heat shock protein increase.

**Conclusion/Significance:**

Intracerebroventricular injection of GLP-1 MSC capsules shows neuroprotective potential in the SOD1 (G93A) mouse model.

## Introduction

Amyotrophic lateral sclerosis (ALS) is a fatal neurodegenerative disorder which causes death of motor neurons in the cortex, brain stem and spinal cord. Patients develop rapidly progressive paralysis and muscle wasting and ultimately die due to respiratory insufficiency within 3–5 years after diagnosis [1]. Aproximately 10% of ALS cases are familial (fALS). About 20% of these fALS cases are linked to mutations in the gene coding for superoxide dismutase 1 (SOD1) [2]. In a transgenic mouse model expressing a mutant form of SOD1 carrying the Gly_93_ → Ala substitution, progressive death of motor neurons occurs in the ventral horn region of the lumbar spinal cord and the mice develop a phenotype similar to ALS [3].

So far, only the glutamate antagonist riluzole has shown marginal neuroprotective potential in the treatment of amyotrophic lateral sclerosis (ALS) [4]. Therefore, current efforts focus on experimental therapies. Recent studies have demonstrated that stem cell transplantation presents a novel therapeutic approach for a variety of neurological diseases (for review see [5]). Current attempts to establish cell therapy in ALS mainly focus on the generation of a more protective environment for degenerating motor neurons rather than on cell replacement. Genetic modification of transplanted cells aiming to increase the production of trophic factors is feasible and enhances the benefit of native cells [6]–[8]. The use of a single drug targeting only one out of many interacting pathomechanisms in ALS has proven to be of little or no effect in a large number of previous studies. Therapeutic approaches which combine several mechanisms of action appear more promising.

In this context, glucagon-like peptide 1 (GLP-1) is an interesting candidate for the treatment of neurodegenerative disorders. GLP-1 is an intestinal peptide, stimulating glucose-dependent insulin secretion after food intake and thereby leading to reduction of blood glucose levels [9], [10]. GLP-1 and its receptor can be found in pancreatic β cells, intestinal cells but also in the brain [11], [12]. Besides its insulinotropic effects, GLP-1 exhibits anti-oxidant capacities [13] and is neuroprotective against excitotoxicity in vitro and in vivo [14], [15]. While GLP-1 penetrates the blood brain barrier, its short half life prevents systemic administration [16], [17]. This led to the development of GLP-1 analogues with increased half life such as exendin 4 or liraglutide [18]–[20] for subcutaneous injection. Several studies showed therapeutic potential of these GLP-1 agonists in animal models of neurodegenerative diseases such as Alzheimer’s disease [21], Parkinson’s disease [22], Huntington’s disease [23] and also after experimental traumatic brain injury [24]. In a study in SOD1 (G93A) mice, exendin 4 did not prolong survival or slow down disease progression of the mice, but led to amelioration of motor neuron [25].

One major advantage of intracerebroventricular implantation of a vehicle releasing GLP-1–as opposed to daily injections of GLP-1 analogues is that it warrants continuous release of the neurotrophic factor. Another method to assure continuous growth factor delivery would be the use of osmotic minipumps which must be replaced every few weeks. Encapsulated cells instead of mini pumps allow the abandonment of catheters which reduces the risk of complications and requires only one surgical procedure that was shown several times to be feasible in humans before [26], [27].

Encapsulated cell biodelivery has been suggested as a novel approach in cellular therapy to deliver therapeutic substances like GLP-1 in a sustainable fashion. Encapsulation in a biopolymer material allows the use of non-autologous cells as it prevents any host versus graft reaction [28].

In our study, we investigated an encapsulated mesenchymal stromal cell (MSC) line, which has been modified to produce GLP-1. Neuroprotective effects of these GLP-1 producing MSC capsules have already been shown in experimental traumatic brain injury and in a transgenic Alzheimer’s disease model [21], [24]. The rationale to use these genetically modified MSC was to combine the genuine neuroprotective potential of adult stem cells together with their ability to serve as a vehicle for continuous release of a trophic factor. In a mouse model of Alzheimer’s’ disease, is has already been established that the neurotrophic effects of native mesenchymal cells can be potentiated by increased GLP-1 production [8].

We administered GLP-1 MSC capsules to transgenic SOD1 (G93A) mice via intracerebroventricular injection before disease onset (day 40). By monitoring of survival, motor function, weight and general condition and by additional histological analyses, we could provide evidence for their neuroprotective potential in the ALS mouse model.

## Results

### Survival Study

#### General condition

**Figure 1:**
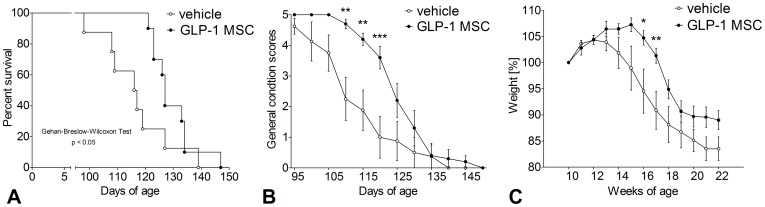
Effects of GLP-1 treatment on survival times, general condition and weight measurements. Presymptomatic GLP-1 treatment (GLP-1 MSC) prolonged survival (A) and improved general condition (B) compared to vehicle controls (vehicle). GLP-1 treated animals gained weight for a longer period than vehicle controls (C). Data are mean ± SEM of 8 (5♀/3♂) (control) and 10 (6♀/4♂) (GLP-1) treated animals. (A) Kaplan-Meyer Curve, followed by Gehan-Breslow-Wilcoxon test. (B & C) two-way ANOVA, followed by Bonferroni post-test. (**) p<0.01; (***) p<0.001.

Disease onset was defined by daily assessment of the general condition of the mice. Animals treated with GLP-1 MSC showed a slighter deterioration compared to vehicle controls (figure 1B). The shift in symptom onset was 15 days on average. Differences to controls were significant between day 110 and 120 (Two-way ANOVA, p<0.01 and p<0.001).

#### Survival

Survival times of animals treated with GLP-1 MSC were significantly prolonged as compared to vehicle-treated mice (figure 1A) (Gehan-Breslow-Wilcoxon test, p<0.05). On average, GLP-1 MSC- treated animals lived 13 days longer than the controls (130d vs. 117d).

#### Weight

Treatment with GLP-1 MSC led to a significant delay of weight loss (figure 1C). While vehicle treated controls started to lose weight already at week 14, GLP-1 MSC- treated animals gained weight until week 15. Significant differences to controls were measured for two weeks (week 16 & 17, two-way ANOVA, p<0.05 & p<0.01).

#### Rotarod

**Figure 2:**
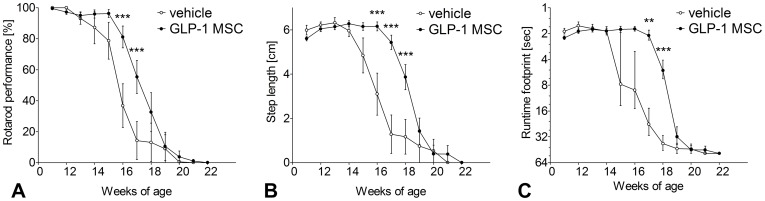
Effects of GLP-1 treatment on rotarod performance and footprint analyses. Presymptomatic GLP-1 treatment (GLP-1 MSC) improved rotarod performance (A), step length (B) and runtime (C) compared to vehicle controls (vehicle). Data are mean ± SEM of 8 (5♀/3♂) (control) and 10 (6♀/4♂) (GLP-1) treated animals. Two-way ANOVA, followed by Bonferroni post-test. (*) p<0.05; (**) p<0.01; (***) p<0.001.

Rotarod performance of vehicle treated controls started to decrease at week 13, while performance of GLP-1 MSC- treated animals was not altered before week 16 (figure 2A). For two weeks the deterioration of motor performance was significantly less in the GLP-1 MSC group (week 16 & 17, two-way ANOVA, p<0.001).

#### Footprint analyses – step length

First decrease in step length occurred at week 14 in vehicle treated controls; therefore 3 weeks earlier than in GLP-1 MSC- treated mice (figure 2B). Differences between both groups were significant from week 16 to week 18 (Two-way ANOVA, p<0.001).

#### Footprint analyses – runtime

While runtime along the footprint track started to increase not before week 18 in the GLP-1 MSC- treated group, vehicle treated controls already showed an increase at week 15 (figure 2C). From week 17 to week 18 these differences became significant (Two-way ANOVA, p<0.01 & p<0.001).

### Histological Analyses

**Figure 3:**
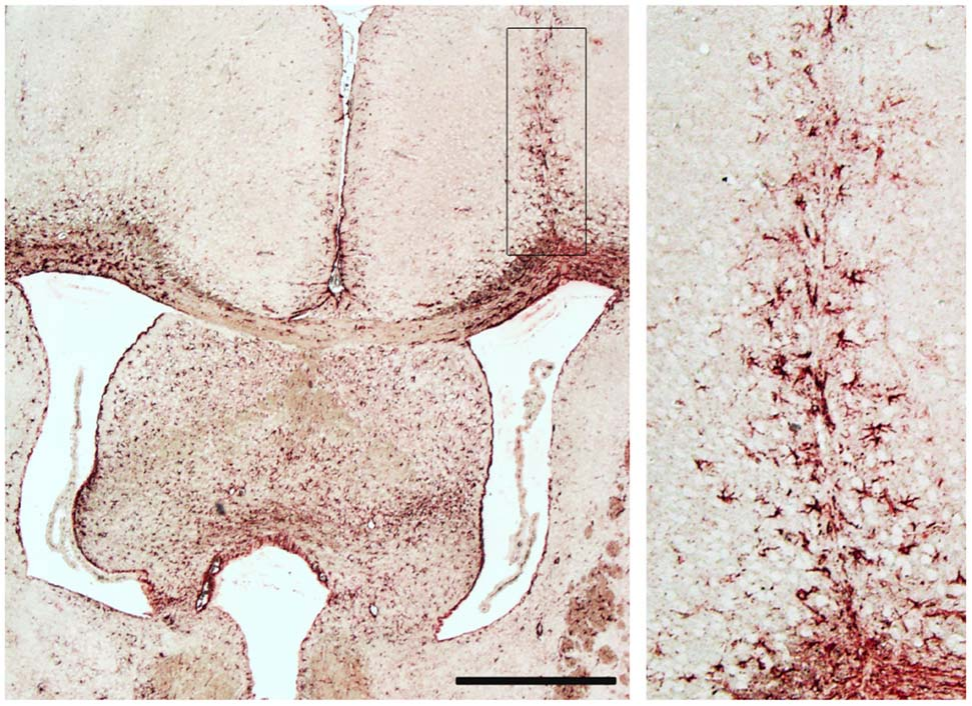
Analysis of post mortem brain tissue at day 110 of SOD1 (G93A) mice. In 5 of 12 animals there was a glial scar visible above the right ventricle as it was shown by GFAP staining. Rectangle in the left picture is showed enlarged on the right side. Scale bar 500 µm.

Brain sections of 6 vehicle and 6 GLP-1 MSC- treated mice were studied by GFAP staining. We found a glial scar above the right ventricle in five of 12 animals (figure 3). Capsules within the ventricles or in surrounding tissue were not detectable as most likely the capsules remained in the ventricles and were washed out during perfusion of animals prior to paraffin embedding. Enlarged ventricles were not observed.

**Figure 4:**
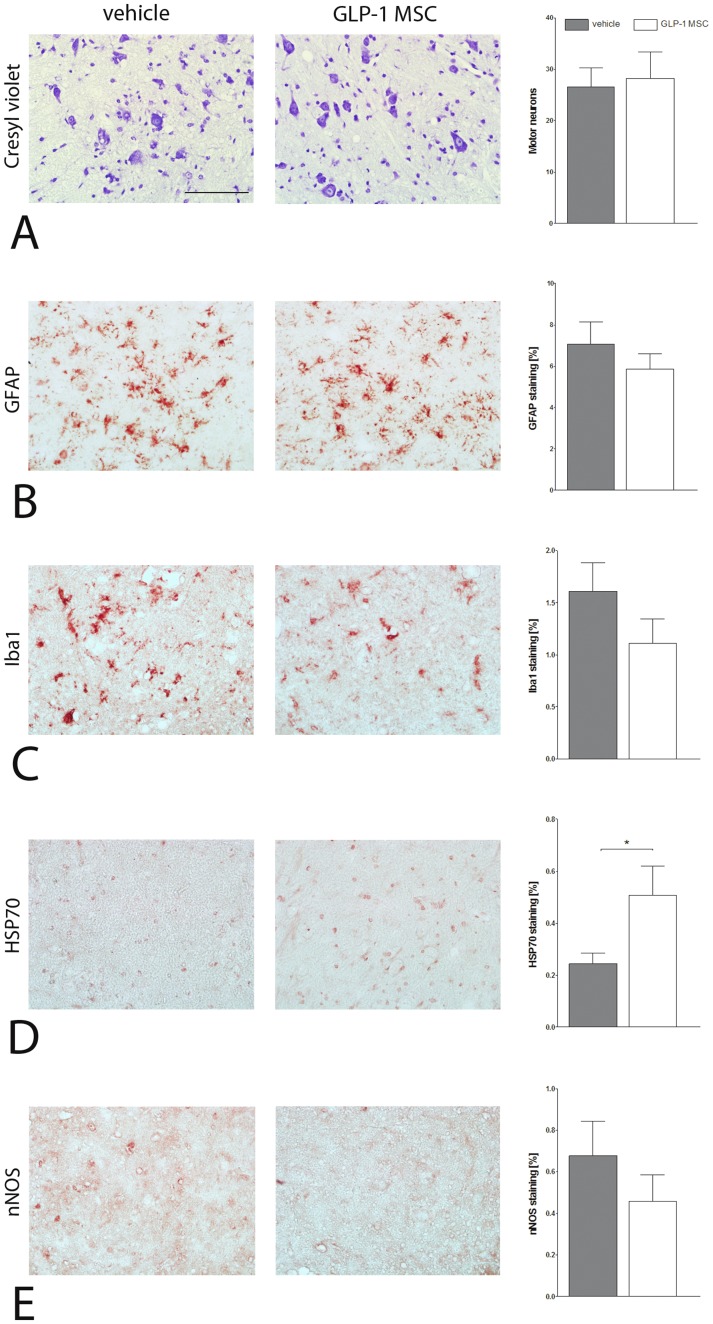
Immunohistological analysis of spinal cord tissue at day 110 of SOD1 (G93A) mice. (A) Motor neuron loss was not ameliorated by GLP-1 treatment (GLP-1 MSC) compared to vehicle treated controls (vehicle). (B) Astrocytosis (C) and microglial activation were marginally less in GLP-1 treated animals compared to controls. (D) Heat shock response was significantly increased by GLP-1 treatment and nNOS (E) was reduced slightly in these animals. Unpaired t-test. (*) p<0.05. Scale bar 100 µm.

Motor neuron numbers were not significantly altered between vehicle- and GLP-1 MSC- treated groups as shown by cresyl violet staining (figure 4A). Treatment with GLP-1 MSC led to a reduction of astrocytosis as detected by GFAP (glial fibrillary acidic protein) staining in lumbar spinal cord sections compared to vehicle treated controls even though quantitative analysis was not statistically significant (figure 4B). Similarly, microgliosis as measured by Iba1 (ionized calcium binding adaptor molecule 1) immunostaining was reduced in GLP-1 MSC- treated animals (figure 4C). Staining for anti-HSP70 (heat shock protein 70) revealed a significantly increased heat shock response in GLP-1 MSC- treated mice compared to controls (unpaired t-test, p<0.05) (figure 4D). nNOS (neuronal nitric oxide synthase) immunolabelling was slightly decreased in the GLP-1 MSC- treated animals (figure 4E).

**Figure 5:**
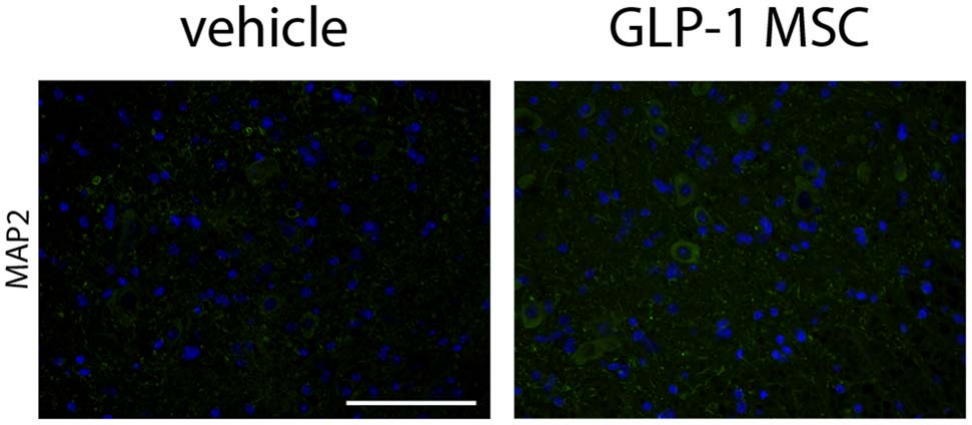
Immunhistological analysis of MAP2 in spinal cord tissue of SOD1 (G93A) mice. Staining against microtubule associated protein 2 was stronger in animals treated with GLP-1 (GLP-1 MSC) compared to vehicle treated controls (vehicle). Scale bar 100 µm.

Even though counting of motor neuron cell bodies in cresyl violet stained spinal cord sections did not result in apparent differences between GLP-1 MSC and vehicle-treated animals, staining for mictrotubule-associated protein 2 (MAP2) as a neuronal marker shower more intense labelling, namely of neuronal processes in spinal cord sections of GLP-1 MSC- treated animals (figure 5).

**Figure 6:**
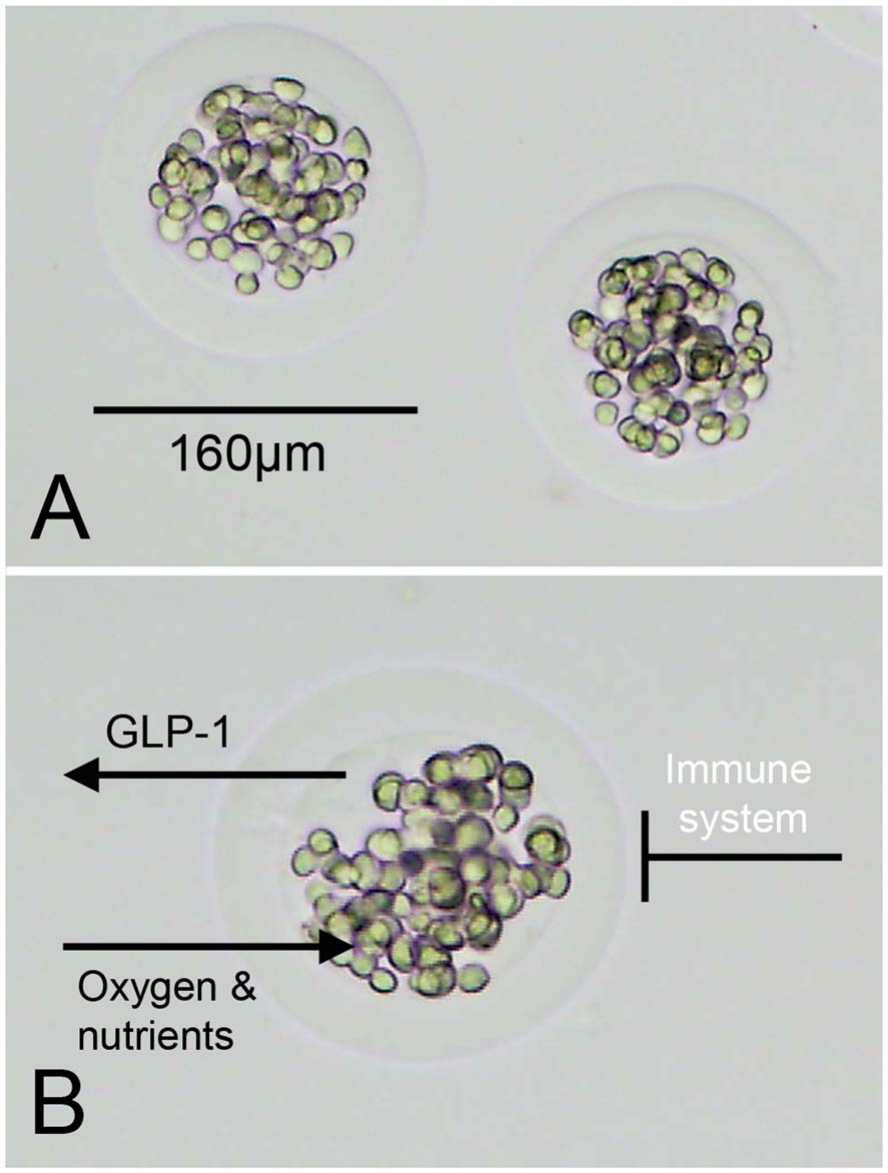
Alginate encapsulated GLP-1 producing mesenchymal cells. (A) Encapsulation with alginate leads to GLP-1 MSC capsules with a mean diameter of 161 µm. (B) GLP-1, oxygen and nutrients are able to pass the alginate barrier, but the cells are protected against the hosts’ immune system.

## Discussion

Cellular therapy is currently being investigated as a novel therapeutic option for the treatment of ALS, mainly with the aim to provide a neuroprotective environment and trophic support for degenerating motor neurons. Mesenchymal stem cells (MSC) present good candidates for this approach as they are easily available, highly proliferative and barely immunogenic [29]. A previous study assessed the effect of intraspinal injection of MSC in an ALS rodent model: the authors showed moderate improvements in motor function and survival which were attributed to anti-inflammatory and neurotrophic effects of MSC [30]. One reason for the limited efficacy of intraspinal injection of cells in ALS could be that motor neuron degeneration is widespread along the spinal cord, brain and brainstem [1]. As cells do barely migrate following injection into brain or spinal cord parenchyma, local administration may therefore not be sufficient to delay disease progression over a long period. Other studies have evaluated the benefit of intravenous administration of mesenchymal cells in ALS mouse models and could show that cells migrated some distance away from blood vessels to the gray and white matter of brain and spinal cord [31], [32]. But intravenous administration holds the risk of cell loss during circulation due to settling of infused cells in many peripheral tissues, such as lung, liver or spleen [32], [33], and moreover requires high numbers of cells.

As opposed to local intraspinal or intravenous administration, intracerebroventricular injection has the advantage that neuroprotective factors released by the cells can directly reach the whole spinal cord and brain via the cerebrospinal fluid (CSF), reducing the risk of adverse effects and the number of cells required. This is a major advantage for future translation into clinical trials. It is not necessary to make the cells enter the spinal cord directly because trophic factors can be distributed via the CSF. This assumption is confirmed by a study of Zhang et al. who showed limited migration of intrathecally administered human MSC in the spinal cord parenchyma of ALS mice but nevertheless significant neuroprotection [34].

There is evidence that MSC cause direct trophic effects without further differentiation into new phenotypes, probably due to release of trophic factors such as VEGF and BDNF [35], [36]. To further enhance the effects of native stem cells, they can be genetically engineered to produce neuroprotective proteins. This approach was tested before in studies using genetically modified GDNF-releasing MSC in a rat model of ALS: The protective effects of native MSC on motor neuron survival, preservation of motor endplates and survival and motor function of animals were improved by GDNF producing MSC [7]. Encapsulation is a promising approach to reduce the risks and complications associated with cellular therapies in the central nervous system: In a first phase I clinical trial in ALS to assess safety and tolerability of encapsulated genetically engineered baby hamster kidney (BHK) cells releasing human ciliary neurotrophic factor (CNTF), therapeutic levels of the secreted peptide could be detected for several weeks without limiting side effects [37]. A phase I/II clinical trial confirmed the safety and tolerability of intrathecal implants of these cell capsules, with signs of only a very mild humoral immune response [38]. Studies in experimental animal models using the same cell capsules as in the present study showed complete prevention of any immune response by microencapsulation of cells prior to transplantation [21], [24]. Use of encapsulated cells may further be considered as a safety measure against tissue damage which occurred in a study assessing intraspinal transplantation of non-encapsulated cells in a model of focal spinal cord demyelination: MSC migrated into the tissue and caused collagen depositions and subsequent local tissue damage [39]. Our post mortem analysis of brain sections after intracerebroventricular injection of encapsulated MSC showed only minor glial scars along the injection channel in part of the animals but no further abnormalities such as enlargement of the ventricles. This indicates safety and tolerability of the implantation technique. In the present study, the intestinal peptide GLP-1 was used to increase the therapeutic capacity of MSC, based on several studies showing neuroprotective effects of a stimulation of GLP-1 receptors which are present in the mammalian brain [14], [21], [24], [40]. While analogues of GLP-1, like exendin 4 or liraglutide, showed neuroprotective potential in models of Alzheimer’s [20] and Parkinson’s disease [19], [41], the effect in ALS mice so far has been insufficient. Exendin 4 treatment via subcutaneous pumps protected motor neurons and reduced GFAP and caspase 3 amount in spinal cord tissue of SOD1 mice, but disease progression and survival of the animals were not affected [25].

We decided to use GLP-1 releasing MSC to combine the protective effects of adult stem cells and continuous growth factor delivery into the CSF. The commercial availability of well characterized GMP manufactured GLP-1 producing MSC allows fast translation to clinical trials in case of a positive outcome in preclinical studies. Previous studies with GLP-1 releasing CellBeads®, have confirmed steady GLP-1 production at a rate of 2.85–3.68 fmol/capsule/h after intracerebroventricular injection. Further it was shown that GLP-1 reaches the affected regions by circulation within the CSF after intracerebroventricular injection in cats [40] and rats [24] by measurement of the CSF concentrations of GLP-1. These studies have also proven steady GLP-1-release of CellBeads® after explantation and a cell viability of at least 95%, independent from the time interval between cell implantation and analysis [24]. Based on these results, we conclude that the beneficial effects of intracerebroventricular injection of GLP-1 producing MSC capsules in SOD1 (G93A) mice resulted at least in parts from increased GLP-1 concentrations in the CSF. Treatment with GLP-1 releasing MSC significantly increased lifespan and improved motor function of SOD1 (G93A) mice. Even though motor neuron loss as assessed by cresyl violet staining and counting of motor neuron cell bodies was not significantly different between vehicle- and GLP-1 MSC-treated groups, we found a stronger signal of MAP2 staining after GLP-1 treatment. One of the earliest events in mutant SOD1 mice is impaired axonal transport [42], [43]. Increased staining for could indicate preservation of neuronal processes, resulting in increased functional capacity of remaining motor neurons due to preserved integrity of microtubules and axonal transport. Further experiments should include analysis of neuromuscular end plates to clarify whether GLP-1 MSC have an effect on terminal motor axons. Besides a direct effect on motor neurons, positive effects on the disease course may be explained by an anti-inflammatory mechanism of action of GLP-1-releasing MSC. Increased astrocytosis and microglial activation are hallmarks of the disease [44] and is now well recognized that SOD1 (G93A) glial cells contribute to motor neuron death in ALS [45], [46]. Intracerebroventricular injection of GLP-1 releasing MSC led to a (not significant) reduction of astrocytosis and to a significant increase of the heat shock response, suggesting less functional impairment of remaining astrocytes. Increased levels of HSP were already shown to be neuroprotective [47] and the HSP-inducing drug arimoclomol slowed down disease progression and increased survival in ALS mice [48]. This is in line with the reduced microglial activation and nNOS immunostaining after GLP-1 MSC injection. Similar to astrocytes, unaffected microglia provide trophic support but activation leads to increased release of neurotoxic factors such as nitric oxide [49], [50]. Reduced microglial activation therefore may lead to reduced levels of nitric oxide and therefore have contributed to the better outcome of GLP-1 MSC-treated mice.

In our pilot study we could demonstrate the feasibility of intracerebroventricular injection of encapsulated GLP-1 producing MSC in ALS mice. Our results regarding motor performance and survival of the animals as well as the observed effects on inflammatory markers in the spinal cord strongly suggest further evaluation of the potential of encapsulated MSC therapy for treatment of ALS.

## Material and Methods

### Ethics Statement

All experiments were carried out in strict accordance with the internationally accepted principles in the care and use of experimental animals and were approved by the Institutional Animal Care and Research Advisory Committee at Hanover Medical School and permitted by the Lower Saxonian State Office for Consumer Protection and Food Safety regional (Permit Number: AZ 07/1324).

### Animals

G93A transgenic familial ALS mice (high copy number; B6SJLTg (SOD1-G93A)1Gur/J) [3] were obtained from The Jackson Laboratory (Bar Harbor, ME, USA). These mice over-express the human mutant SOD1 allele containing the Gly93 → Ala (G93A) substitution. We maintained the transgenic G93A hemizygotes by mating transgenic males with B6SJLF1/J hybrid females. Transgenic offspring was genotyped by PCR assay of DNA obtained from tail tissue. Mice were housed under controlled conditions (12∶12 light:dark cycle) with free access to food and water. Animals of the same sex were kept in groups of up to five animals in Makrolon cages type II (UNO, Zevenaar, Netherlands). Males were kept solitary in the same cage type only when they were also used for breeding.

### Alginate Microcapsules

A human, bone marrow-derived, single cell derived mesenchymal stromal cell line provided by CellMed AG, Alzenau, Germany as previously described [21] was used in this study. It was immortalized by transduction with the human Telomerase Reverse Transcriptase (hTERT) gene [51]. After transfection with a plasmid vector encoding a GLP-1 fusion gene, the cells produced an 8.7 kDa dimeric GLP-1 molecule. The cells are embedded in a spherical shaped alginate matrix (about 160 µm in diameter, figure 6). The alginate matrix is generated by cross-linking alginate with barium ions. Each capsule (trademark CellBeads®) contained about 94 cells. Until use, the cell capsules were stored in liquid nitrogen.

### Preoperative Procedure

For surgery animals were anesthetized by a combination of ketamine (0.1 ml/100 g, 100mg/kg), xylazine (0.01 ml/100g, 2 mg/kg) and midazolame (0.05 ml/100 g, 0.5 mg/kg), prepared under sterile conditions with 0.9% sodium chloride. Appropriate to the body weight (0.1 ml/10 g) anaesthesia was administered intraperitoneally. Duration of anaesthesia was up to 60 minutes, which was sufficient for the surgery. Depth of anaesthesia was controlled by the toe-and eyelid- reflex.

### Cerebroventricular Injection

The heads of the animals were shaved and disinfected. Eye ointment protected the eyes against dehydration. The head was fixed with ear bars in a stereotactic frame and the skin was disclosed longitudinally. Bregma was visualized by 30% hydrogen peroxide and coordinates of bregma were recorded. From bregma injection coordinates (1 mm lateral, 0.5 mm anterior) were adjusted before the cranial bone was enclosed under visual control with a drill head of 1.4 mm diameter. Following the preparation of injection position, 100 µl of the cell solution was dropped on a glass dish and up to 30 GLP-1 MSC capsules were taken with a 10 µl Hamilton syringe under visual control. Thy syringe filled with GLP-1 MSC capsules was kept upright until usage so the beads were concentrated in the top of the tip. After adjustment of injection coordinates, the syringe was inserted 5 mm into the brain, and then withdrawn 1 mm so the cells were injected 4 mm distal from the cranial bone. 1 µl cell solution was injected all at once. After a waiting period of 3 minutes the syringe was withdrawn slowly and washed with 0.9% sodium chloride under visual control. Remaining cell capsules were counted and recorded in the surgery protocol.

### Postoperative Procedure

Following wound closure animals received a single dose of carprofene (5 mg/kg subcutaneously) and metamizole via drinking water (200 mg/kg/day) for 3 postoperative days for analgesia.

### Treatment Groups

Mice were treated with either GLP-1 producing MSC capsules or empty alginate capsules as vehicle control (control: n = 10 (6♀/4♂); GLP-1 MSC: n = 8 (5♀/3♂) for assessment of survival and motor performance; control: n = 6 (5♀/1♂); GLP-1 MSC: n = 6 (5♀/1♂) for histological analyses). An increase in the therapeutic effect of encapsulated GLP-1 producing MSC capsules as compared to native encapsulated MSC has already been proven before [24]. To reduce the total number of animals in the study, we therefore did not include a second control group of mice injected with alginate beads filled with native MSC. Animals of each litter were randomly attributed to either the GLP-1 MSC group or the control group and were monitored until they reached disease end stage. The cerebroventricular injection of either GLP-1 producing or empty capsules was administered once at a presymptomatic disease stage (d40). After surgery, experimenters were blinded to treatment groups so that animals were objectively monitored throughout the whole period of behavioural assessment.

### Behavioural Assessment

#### General condition

For weekly assessment of general condition from week 14 we used a behavioural score system as previously described [52], [53], based on the score developed by Vercelli et al. [30] from 1 to 5 defined as follows:

1. healthy without any symptoms of paralysis,
2. slight signs of destabilized gait and paralysis of the hind limbs,
3. obvious paralysis and destabilized gait
4. fully developed paralysis of the hind limbs, animals only crawl on the forelimbs
5. fully developed paralysis of the hind limbs, animals predominantly lie on the side and/or are not able to straighten up after turning them on the back or lost more than 20% of their starting weight.

When animals reached a score of 2, macerated food was given daily for easy access to food and hydration. Reaching a score of 1, the animals were euthanized.

#### Evaluation of onset and survival

Day of onset was set as the first day the animals reached a score of 4 in the daily behavioural assessment. Animals were killed when they reached a score of 1 and this age (in days) was recorded as survival time.

#### Weight

We recorded the weight of the animals weekly, beginning at 10 weeks of age, using a normal digital balance ranging up to 800 g in 0.1 g steps.

#### Rotarod

Beginning at 11 weeks of age, we analyzed motor function using a rotarod apparatus from IITC (IITC Life Science Inc. California). After an adaptation period of 5 days the test was performed weekly. Mice had to remain on the rotating cylinders for up to 180 s with an increasing speed up to 18 rpm. Rotarod test was performed as previously described [52].

#### Footprint analyses

Footprint analyses for step length and runtime were performed weekly starting the same week as rotarod tests performance [52]. The hind feet were dipped into black finger print and animals were placed on a gangway covered with conventional masking tape. Footprints were analyzed with respect to the step length using the FOOTPRINTS software (Version 1.22 by K. Klapdor and B. Dulfer [54]). In addition, the time animals needed to run along the track (50 cm) was measured.

### 2.4 Histological Evaluation

At d110, six vehicle and six GLP-1 MSC-treated animals (5♀/1♂) were sacrificed by an overdose of anesthetic (ketamine (0.1 ml/100 g, 100 mg/kg), rompune (0.01 ml/100 g, 2 mg/kg) and midazolame (0.05 ml/100 g, 0.5 mg/kg)). After transcardial perfusion with 25 ml 4% paraformaldehyde (PFA) in phosphate buffer (PBS), the brain and lumbar part of the spinal cord was removed. Postfixation for 1 day in 4% PFA was followed by storage in 70% ethanol until dehydration by progressively more concentrated ethanol baths and xylene (Mallinckrodt Baker B.V., Deventer, Netherlands) and embedding in paraffin blocks. Sections of 7 µm were cut on a microtome and 5–6 sections were transferred to one object slide, respectively.

#### 2.4.1 Motor neuron survival

One slide per animal (i.e. 5–6 spinal cord sections) was rehydrated in xylene and graded ethanol, stained with 0.2% Thionin, dehydrated in graded ethanol and xylene and coverslipped with Eukitt quick hardening mounting medium (Sigma-Aldrich, Steinheim, Germany). At 20x magnification in an Olympus B×61 microscope, cells in the ventral horn region with a diameter >200 µm^2^ were defined as motor neurons, according to Chen et al., who determined α- motor neurons as cells with cell body areas ranging from 200 to 1100 µm^2^ [55]. Motor neurons were counted, using cell* software (Olympus, Hamburg, Germany). Only intact ventral horn regions were used for counting so that, depending on the quality of the section, 5 to 12 ventral horn regions were counted for motor neuron numbers and the mean was used for statistical evaluation.

#### 2.4.2 Immunohistochemistry

For immunohistochemical staining, slides were rehydrated in xylene, graded ethanol and PBS. For antigen retrieval, slides were boiled for 5–10 min in citrate buffer and cooled for 15 minutes at 4°C. After immersion in PBS, slides were blocked for 5 min with peroxidase block (DakoCytomation, Glostrup, Denmark) and antibody diluent (DakoCytomation, Glostrup, Denmark) for 1 h followed by overnight incubation at 4°C with primary antibodies specific for GFAP (1∶600; polyclonal rabbit anti glial fibrillary acidic protein; DakoCytomation, Glostrup, Denmark), Iba1 (1∶250; polyclonal rabbit anti ionized calcium binding adaptor molecule 1; WAKO Chemicals GmbH, Neuss, Germany), HSP70 (1∶250; monoclonal mouse anti heat shock protein; Acris antibodies, Herford, Germany) or nNOS (1∶250; polyclonal rabbit anti neuronal nitric oxide synthase; Millipore, Bedford, USA) diluted in antibody diluent. Secondary HRP antibody anti-rabbit or anti-mouse respectively (EnVision+System-HRP (AEC+); DakoCytomation, Glostrup, Denmark) was added for 30 min, followed by 20–25 min incubation in chromogene substrate (EnVision+System-HRP (AEC); Dako-Cytomation, Glostrup, Denmark). Slides were covered with Kaiser’s glycerol gelatin (Merck, Darmstadt, Germany).

For quantitative analyses of astrocytosis, microglial activation, heat shock response and nNOS staining, the percentage of stained area of the ventral horn was determined using the phase analysis tool of cell* software in pictures taken at 20x magnification by an Olympus Bx61 microscope.

For MAP2 staining, slides were blocked with 5% goat serum in PBS with 0.3% Triton X-100 (Sigma-Aldrich, Steinheim, Germany) for 1 h followed by overnight incubation at 4°C with primary antibodies specific for MAP2 (1∶250; polyclonal rabbit anti microtubule-associated protein 2; Millipore, Bedford, USA) diluted in 5% goat serum in PBS with 0.3% Triton X-100. Secondary anti-rabbit or Alexa Fluor 488 antibody (1∶500; anti-IgG (H+L); Invitrogen, Darmstadt, Germany) was added for 45 min. Slides were then counterstained with the fluorescent DNA dye DAPI (10 mg/ml; Invitrogen, Darmstadt, Germany).

#### Statistics

Data were analyzed by two-way ANOVA in order to evaluate time evolution of the different parameters. In case of significant parameter interaction (p<0.05), comparison with a Bonferroni posthoc test was performed. All data are presented as mean ± SD and significance level was set as p<0.05. Survival was analyzed by the Gehan- Breslow- Wilcoxon test with a significance level of p<0.05.
